# The effect of tooth brushing, irrigation, and topical tetracycline administration on the reduction of oral bacteria in mechanically ventilated patients: a preliminary study

**DOI:** 10.1186/s12903-016-0224-x

**Published:** 2016-06-07

**Authors:** Saki Hayashida, Madoka Funahara, Motohiro Sekino, Noriko Yamaguchi, Kosuke Kosai, Souichi Yanamoto, Katsunori Yanagihara, Masahiro Umeda

**Affiliations:** Department of Clinical Oral Oncology, Nagasaki University Graduate School of Biomedical Sciences, 41-7-1 Sakamoto, Nagasaki, 852-8588 Japan; Division of intensive care, Nagasaki University Hospital, 41-7-1 Sakamoto, Nagasaki, 852-8588 Japan; Department of Laboratory Medicine, Nagasaki University Graduate School of Medicine, 41-7-1 Sakamoto, Nagasaki, 852-8588 Japan

**Keywords:** Ventilator-associated pneumonia, Oral bacteria, Oral care, Irrigation, Topical antibiotic administration

## Abstract

**Background:**

One of the main causes of ventilator-associated pneumonia (VAP) is thought to be aspiration of oropharyngeal fluid containing pathogenic microorganisms. The aim of this study was to examine the effects of various oral care methods on the reduction of oral bacteria during intubation.

**Methods:**

First, the effect of mechanical oral cleaning was investigated. The bacterial count on the tongue and in the oropharyngeal fluid was measured after tooth brushing, irrigation, and three hours after irrigation in mechanically ventilated patients at the intensive care unit (ICU).

Next, the efficacy of topical administration of tetracycline and povidone iodine on the inhibition of bacterial growth on the tongue and in the oropharyngeal fluid was examined in oral cancer patients during neck dissection.

**Results:**

The number of bacteria in the oropharyngeal fluid was approximately 10^5^–10^6^ cfu/mL before surgery, but increased to 10^8^ cfu/mL after intubation. Oral care with tooth brushing and mucosal cleaning did not reduce oral bacteria, while irrigation of the oral cavity and oropharynx significantly decreased it to a level of 10^5^ cfu/mL (*p* < 0.001). However, oral bacteria increased again to almost 10^8^ cfu/mL within three hours of irrigation.

Oral bacteria did not decrease by topical povidone iodine application. In contrast, 30 min after topical administration of tetracycline, the number of oral bacteria decreased to 10^5^ cfu/mL, and remained under 10^6^ cfu/mL throughout the entire experimental period of 150 min.

**Conclusions:**

While the present studies are only preliminary, these results indicate that irrigation of the oral cavity and oropharynx followed by topical antibiotic administration may reduce oral bacteria in mechanically ventilated patients.

**Trial registration:**

UMIN000018318, 1 August 2015.

## Background

Ventilator-associated pneumonia (VAP) is one of the major complications in the intensive care unit (ICU). It is reported that VAP occurs in 8 to 28 % of mechanically ventilated patients [1–6]. Several risk factors for VAP have been identified, and some methods of prevention have been explored. The main cause of VAP is thought to be due to aspiration of oral bacteria, such as *Staphylococcus aureus*, *Streptococcus pneumonia*, or gram-negative rods [7–10]. Within 48 h of admission to the ICU, oral flora of critically ill patients undergoes a change to predominantly gram-negative flora that includes more virulent organisms [11–13].

0.12 % chlorhexidine is widely applied in the oral cavity to prevent VAP [14]. However, use of this agent on the mucosal surface is prohibited in Japan due to case reports of anaphylaxis. One of the main causes of VAP is thought to be due to aspiration of oropharyngeal fluid containing pathogenic microorganisms. Many investigators have attempted to reduce VAP by various oral care methods. Although some investigators have attempted to reduce VAP by tooth brushing, the results of three randomized controlled trials (RCTs) [11, 15, 16] showed no beneficial effect of tooth brushing on the prevention of VAP. The oral care methods utilized in these studies included tooth brushing, swabbing of the palate and tongue, and suction, yet irrigation of the oral cavity and oropharynx with water was not performed. Others attempted to reduce VAP by topical administration of various agents, such as tobramycin, amphotericin B, polymyxin E, gentamycin, colistin, and vancomycin, but could not show the effects on the overall survival [12, 17, 18].

There have been no previous studies focused on the number of oral bacteria in patients during intubation. The aims of this study were to examine the quantitative change of oropharyngeal bacteria after tooth brushing, irrigation and topical application of antiseptic or antibiotics.

## Methods

### Oral care in mechanically ventilated patients

#### Patients

The study consisted of 45 patients (31 males, 14 females) of an average age of 65 years (range, 36–87 years) with mechanical ventilation by oral intubation who received oral care by dentists and dental hygienists at the ICU of Nagasaki University Hospital from January to September, 2014. These patients consisted of 21 elective operations and 24 emergent admissions. Causes for hospitalization in the ICU was heart disease in 22 patients, infection (septic shock) in 14 patients, cardiopulmonary arrest in 6 patients, organ transplantation in 2 patients, and perforation of the vein in a leg in one patient. Patients received dental treatment, including tooth extraction, temporary splints, and root canal treatment, except those of emergent admissions.

#### Oral care methods

Oral care by dental hygienist was performed the day before surgery except for patients who had had urgent hospitalization in the ICU. The methods of oral care were: removal of dental plaque with tooth brush, interdental brushing and dental flossing, tooth polishing, removal of tongue coating, and gargling.

After hospitalization in the ICU, patients underwent oral care by dentists and dental hygienists. First, the tape that secured the tracheal tube was removed, and tooth brushing, interdental brushing, and cleaning of the tongue and mucosal surface with a sponge brush or wet tissue for mouth care was performed using simultaneous suction. Next, irrigation of the oral cavity and oropharynx with 200 ml water was performed.

#### Evaluation

Dental plaque, macroscopic appearance of the tongue coating, and number of oral bacteria were examined the day before surgery and the next day after surgery. Dental plaque was evaluated by the Debris Index-Simplex (DI-S) of the Oral Hygiene Index-Simplex (OHI-S) in Table 1 [19]. Quantities of plaque on the upper and lower first molars on both sides, the right upper central incisor, and the left lower central incisor were macroscopically examined. Quantity of tongue coating was classified into four categories by the method previously reported by Kojima in Table 2 [20].

**Table 1:** OHI-S(DI-S)

Score	Examination criteria
0	No adhesion of plaque
1	Adhesion of plaque within 1/3 of dental crown or regardless of the range exogenous pigmentation
2	Adhesion of plaque within 1/3 ~ 2/3 of dental crown
3	Adhesion of plaque more than 2/3 of dental crown

Calculation method: Total score of tested 6 teeth ÷6

Case of loss of tested tooth: Case of loss of the first molar, substitute by the second molar

**Table 2:** Classification of degree of adhesion of tongue coat adhesion by method of Kojima

First-degree	1/3 dorsum of the tongue and thin
Second-degree	2/3 dorsum of the tongue and thin or 1/3 dorsum of the tongue and thick
Third-degree	2/3 or more dorsum of the tongue and thin or 2/3 dorsum of the tongue and thick
Fourth-degree	2/3 or more dorsum of the tongue and thick

The number of bacteria was measured on the dorsum of the tongue and in the oropharyngeal fluid by the Rapid Oral Bacteria Quantification System (Panasonic Healthcare Co. Ltd., Osaka, Japan) using the dielectrophoresis and impedance measurement method [21, 22]. Because the detection limit of this machine is 10^5^ cfu/mL, actual bacterial counts less than this limit were displayed as 10^5^ cfu/mL. Oral bacteria was measured the day before surgery and the next day after surgery. In the ICU, measurements were performed 1) before oral care, 2) after brushing and mucosal cleaning, 3) after irrigation, and 4) three hours after oral care.

#### Statistical analysis

The differences in DI-S and Tongue Coating Indexes before and after intubation, and in number of bacteria before and after oral care the day before surgery were analyzed statistically using Mann-Whitney’s *U* Test. Further, in the ICU, those before oral care, after brushing and mucosal cleaning, after irrigation, and three hours later were also analyzed by Kruskal-Wallis test.

### Topical administration of povidone iodine or tetracycline

#### Patients

Fifteen patients with oral cancer who underwent neck dissection under general anesthesia were enrolled in the study. After intubation, the oral cavity and oropharynx were irrigated sufficiently with 500 ml of saline. In all patients, 1500 mg of ampicillin/sulbactam was administered parenterally at the start of surgery. The patients were divided into three groups: 1) control group (5 patients), 2) povidone iodine group (5 patients), and 3) tetracycline group (5 patients).

#### Treatment and evaluation

In the povidone iodine group, 5 ml of 10 % povidone iodine solution was applied in the oral cavity after irrigation. The tetracycline group received topical administration of 10 g of 3 % tetracycline ointment on the dorsum of the tongue. The number of bacteria on the dorsum of the tongue and in the oropharyngeal fluid was measured every 15 min by the same method of the first examination until neck dissection was finished. Additionally, the concentration of tetracycline in the oropharyngeal fluid was measured at five minutes, one and two hours after topical administration by bioassay. Statistical analysis was not done because of a small number of patients.

## Results

### Oral care in mechanically ventilated patients

**Fig. 1:**
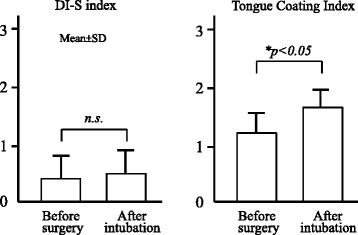
Changes of DI-S and Tongue Coating Indexes before and after intubation

The average DI-S index before surgery and after intubation was 0.33 and 0.47, respectively. There was no increase of DI-S index the next day after intubation, due possibly to adequate perioperative oral management in our hospital. In contrast, the tongue coating index increased the next day after intubation. A thin coat was present in most cases, but this was easily removed by swab (Fig. 1).

**Fig. 2:**
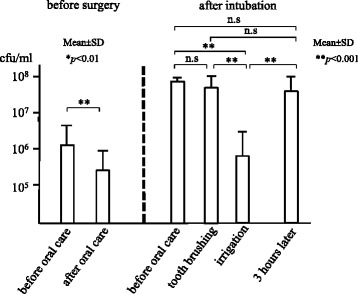
Number of bacteria in the oropharyngeal fluid

**Fig. 3:**
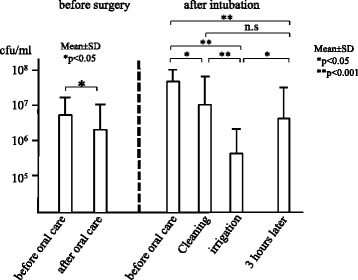
Number of bacteria on the dorsum of the tongue

The number of bacteria in the oropharyngeal fluid before surgery was approximately 10^5^–10^6^ cfu/mL. After hospitalization in the ICU, the bacteria in the oropharyngeal fluid significantly increased compared to before intubation (*p* < 0.001), to approximately 10^8^ cfu/mL. Oral care with tooth brushing and mucosal cleaning didn’t reduce the number of oral bacteria. But irrigation of the oral cavity and oropharynx significantly decreased oral bacteria to a level of 10^5^ cfu/mL(*p* < 0.001). However, three hours after irrigation, oral bacteria significantly increased to almost 10^8^ cfu/mL again (*p* < 0.001) (Fig. 2). The number of bacteria on the dorsum of the tongue showed a result different from the oropharyngeal fluid. Oral care with tooth brushing and mucosal cleaning significantly reduced the number of oral bacteria (*p* < 0.001). Furthermore, irrigation of oral cavity significantly decreased oral bacteria (*p* < 0.001). However, three hours after irrigation, the number of bacteria on the dorsum of the tongue again increased (Fig. 3).

### Topical administration of povidone iodine or tetracycline

**Fig. 4:**
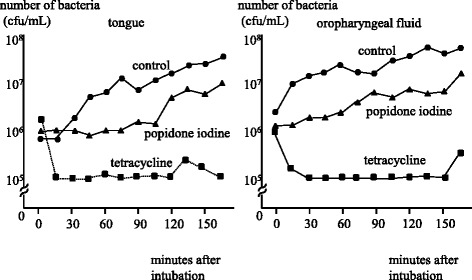
Efficacy of topical povidone iodine and tetracycline

In patients receiving topical administration of povidone iodine, the number of bacteria on the dorsum of the tongue and in the oropharyngeal fluid was slightly decreased compared to the control group. In contrast, topical administration of tetracycline ointment showed remarkable effects on the reduction of bacteria. Thirty minutes after topical administration, the number of bacteria on the dorsum of the tongue and in the oropharyngeal fluid decreased to 10^5^ cfu/mL, and remained under 10^6^ cfu/mL throughout the entire experimental period (Fig. 4).

**Fig. 5:**
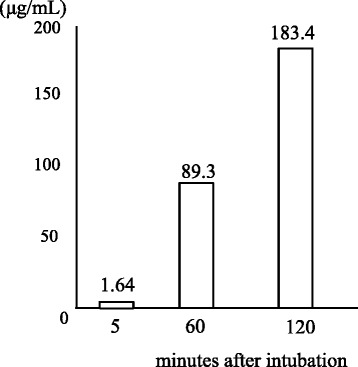
Concentration of tetracycline in the oropharyngeal fluid

The concentration of tetracycline in the oropharyngeal fluid after topical administration on the dorsum of the tongue was 1.70 μg/mL at five minutes, 89.3 μg/mL at one hour, and 183.4 μg/mL at two hours (Fig. 5).

## Discussion

VAP is an airway infection developing more than 48 h after intubation, which is the leading cause of death among hospital-acquired infections. The Institute for Healthcare Improvement (IHI) proposed four prophylaxis for prevention of VAP (VAP bundle) consisting of head-of-bed elevation, a daily “sedation vacation” and a readiness-to-wean assessment, peptic ulcer disease prophylaxis, and deep vein thrombosis prophylaxis. Additionally, a fifth prophylaxis, oral decontamination with chlorhexidine, was added in 2010 [23]. However, oral hygiene practices of tooth brushing, removal of tongue coating, swabbing of oral mucosa, and irrigation were not included in the VAP bundle.

One of the main causes of VAP is thought to be due to aspiration of oropharyngeal fluid containing pathogenic microorganisms. Many investigators have attempted to reduce VAP by various oral care methods. Mori et al. [24] reported that 1252 mechanically ventilated patients who received oral care consisting of swabbing with povidone iodine gargle, tooth brushing, and irrigation with 300 ml of acidic water showed a significantly lower frequency of VAP compared to the 414 patients who did not receive oral care. Sona et al. [25] also described that the implementation of a simple, low-cost oral care protocol of tooth brushing, rinsing with tap water, and subsequent application of a 0.12 % chlorhexidine gluconate chemical solution led to a significantly decreased risk of acquiring VAP. However, their studies were performed with historically controlled patients and therefore the evidence levels were not high. Munro et al. [11], Pobo et al. [15], and Lorente et al. [16] conducted randomized controlled studies of the effect of tooth brushing on the prevention of VAP and concluded that mechanical tooth brushing did not show any significant efficacy. Some investigators attempted topical antibiotic application for the prevention of VAP, but they were unable to find any effect on the improvement of outcome of mechanically ventilated patients [12, 17, 18]. Hillier et al. stated in a literature review that no consensus has been established yet on best practices for oral hygiene in mechanically ventilated patients, although chlorhexidine was the most popular oral care method [14].

The three RCTs of Munro [11], Pobo [15], and Lorente [16] concluded that mechanical oral care was not effective for preventing VAP, as mentioned above. The current preliminary study also demonstrates that tooth brushing and mucosal cleaning with suction had little effect on reducing the number of bacteria in the oropharyngeal fluid. However, tongue and oropharyngeal bacteria decreased significantly after irrigation with 200 ml tap water in the oral cavity and oropharynx. In their RCTs, 0.12 % chlorhexidine was applied in the oral cavity every eight hours. We used 10 % povidone iodine solution and examined its effect on reducing bacteria on the tongue and in the oropharyngeal fluid, because use of 0.12 % chlorhexidine on mucosal surfaces is prohibited in Japan due to a case report of anaphylaxis. As a result, the increase of oral bacteria was slightly inhibited after topical application of 10 % povidone iodine solution.

The number of bacteria on the tongue and in the oropharyngeal fluid significantly decreased to the level of before surgery when irrigation was added after tooth brushing. These findings indicate that irrigation is essential to reduce oral bacteria in mechanically ventilated patients. However, oral bacteria increased again only 3 h after irrigation. We think that the procedures described in the above RCTs were not sufficient to decrease oral bacteria due to the lack of irrigation of the oral cavity and prolonged oral care interval. It has been suggested that mechanical oral care requires irrigation and frequent practice: at least every 3 h.

This study demonstrated that topical administration of tetracycline ointment, an approach different from frequent practice, is an alternative method to reduce oral bacteria. After application of tetracycline ointment on the dorsum of tongue, bacteria both on the tongue and oropharyngeal fluid rapidly decreased to 10^5^ cfu/mL or less, and the effects lasted for at least 150 min. We could not clarify how long the effect lasted, because the surgery finished within 150 min. In contrast, topical administration of povidone iodine showed limited effects on reducing oral bacteria.

Some investigators have reported the effects of oral decontamination on the prevention of pneumonia in ventilated patients. Rodriguez-Roldán et al. [17], applied paste containing tobramycin, amphotericin B, and polymyxin E topically in the oral cavity in 13 ventilated patients, and concluded that nosocomial pneumonia could be prevented by local application of nonabsorbable antibiotics to the oropharynx, although the overall mortality was not improved. Abele-Horn et al. [12] reported that 58 patients receiving topical administration of the same paste demonstrated a decreased incidence of pneumonia compared to the 30 control patients; however, the length of the ICU stay and mortality were similar between the groups. Bergmans et al. [18] also reported that the 92 patients who received topical antimicrobial prophylaxis consisting of an Orabase with gentamycin, colistin, and vancomycin had a reduced frequency of developing pneumonia compared to the 153 patients who did not undergo such a procedure, although this was not associated with shorter duration of ventilation or better survival. Because these authors’ studies failed to demonstrate the efficacy of topical antibiotic administration on the mortality of mechanically ventilated patients, this method has not become a standard treatment.

The present results, demonstrated by bacterial count, showed that tooth brushing and mucosal swab were able to reduce bacteria little in the oropharyngeal fluid, but was significantly decreased after irrigation, and that topical administration of tetracycline ointment on the dorsum of the tongue strongly inhibited the growth of bacteria. The reservoir of microorganisms in the oropharyngeal fluid is not clear. We examined the change of the number of bacteria on the buccal mucosa, palate, dorsum of the tongue, and in the oropharyngeal fluid after intubation, and clarified that bacteria increased rapidly on the tongue and in the oropharyngeal fluid, while that on the buccal mucosa and palate did not during intubation [26]. Some investigators have stated that dental plaque is a reservoir of oropharyngeal bacteria and that removal of dental plaque is important for the prevention of pneumonia in ventilated patients [27, 28]. On the other hand, Penel et al. [29] reported that edentulous patients, who are completely plaque-free, develop surgical site infection (SSI) as frequently as those with teeth in head and neck cancers, which indicated that dental plaque was not a main reservoir of oropharyngeal bacteria. We believe that the surface of the dorsum of the tongue may be one of the reservoirs of intraoral bacteria, and that it is necessary to inhibit bacteria growth on the tongue for prevention of VAP. Our report is unique in that it has demonstrated topical administration of tetracycline on the tongue is able to reduce bacteria in the oropharyngeal fluid, as well as on the tongue. However, before clinical application, we think it is necessary to examine the prevalence of species including resistant bacteria after topical administration of tetracycline, especially in case of prolonged intubation.

## Conclusions

Oral care with irrigation of the oral cavity and oropharynx followed by topical antibiotic administration may reduce oral bacteria in mechanically ventilated patients.

## Abbreviations

DI-S, debris index-simplex; IHI, Institute for Healthcare Improvement; OHI-S, oral hygiene index-simplex; RCTs, randomized controlled trials; SSI, surgical site infection; VAP, ventilator-associated pneumonia; ICU, intensive care unit
